# Within-host whole genome analysis of an antibiotic resistant *Pseudomonas aeruginosa* strain sub-type in cystic fibrosis

**DOI:** 10.1371/journal.pone.0172179

**Published:** 2017-03-08

**Authors:** Laura J. Sherrard, Anna S. Tai, Bryan A. Wee, Kay A. Ramsay, Timothy J. Kidd, Nouri L. Ben Zakour, David M. Whiley, Scott A. Beatson, Scott C. Bell

**Affiliations:** 1 Lung Bacteria Group, QIMR Berghofer Medical Research Institute, Brisbane, QLD, Australia; 2 School of Medicine, The University of Queensland, Brisbane, QLD, Australia; 3 Adult Cystic Fibrosis Centre, Department of Thoracic Medicine, The Prince Charles Hospital, Brisbane, QLD, Australia; 4 Western Australia Adult Cystic Fibrosis Centre, Department of Respiratory Medicine, Sir Charles Gairdner Hospital, Perth, WA, Australia; 5 School of Chemistry and Molecular Biosciences, The University of Queensland, Brisbane, QLD, Australia; 6 Centre for Experimental Medicine, Queen’s University Belfast, Belfast, United Kingdom; 7 Child Health Research Centre, The University of Queensland, Brisbane, QLD, Australia; 8 UQ Centre for Clinical Research, The University of Queensland, Brisbane, QLD, Australia; 9 Pathology Queensland, Microbiology Department, Brisbane, QLD, Australia; Lee Kong Chian School of Medicine, SINGAPORE

## Abstract

A *Pseudomonas aeruginosa* AUST-02 strain sub-type (M3L7) has been identified in Australia, infects the lungs of some people with cystic fibrosis and is associated with antibiotic resistance. Multiple clonal lineages may emerge during treatment with mutations in chromosomally encoded antibiotic resistance genes commonly observed. Here we describe the within-host diversity and antibiotic resistance of M3L7 during and after antibiotic treatment of an acute pulmonary exacerbation using whole genome sequencing and show both variation and shared mutations in important genes. Eleven isolates from an M3L7 population (*n* = 134) isolated over 3 months from an individual with cystic fibrosis underwent whole genome sequencing. A phylogeny based on core genome SNPs identified three distinct phylogenetic groups comprising two groups with higher rates of mutation (hypermutators) and one non-hypermutator group. Genomes were screened for acquired antibiotic resistance genes with the result suggesting that M3L7 resistance is principally driven by chromosomal mutations as no acquired mechanisms were detected. Small genetic variations, shared by all 11 isolates, were found in 49 genes associated with antibiotic resistance including frame-shift mutations (*mexA*, *mexT*), premature stop codons (*oprD*, *mexB*) and mutations in quinolone-resistance determining regions (*gyrA*, *parE*). However, whole genome sequencing also revealed mutations in 21 genes that were acquired following divergence of groups, which may also impact the activity of antibiotics and multi-drug efflux pumps. Comparison of mutations with minimum inhibitory concentrations of anti-pseudomonal antibiotics could not easily explain all resistance profiles observed. These data further demonstrate the complexity of chronic and antibiotic resistant *P*. *aeruginosa* infection where a multitude of co-existing genotypically diverse sub-lineages might co-exist during and after intravenous antibiotic treatment.

## Introduction

Cystic fibrosis (CF) is the most common lethal recessively inherited disease in Caucasians. Respiratory disease secondary to chronic airway infection is the major complication in CF, which causes the majority of mortality and morbidity in patients. *Pseudomonas aeruginosa* is the most common respiratory pathogen isolated from people with CF [1]. Person-to-person transmission of *P*. *aeruginosa* has been documented in CF clinics globally with adverse clinical outcomes correlated with some shared strains [2–5]. In Queensland, Australia, a predominant *P*. *aeruginosa* shared strain, AUST-02, has been detected in patients at six clinics and in 16 clinics nationally (18% of patients with *P*. *aeruginosa* infection) [2,6–8]. We recently reported the emergence of an AUST-02 strain sub-type, M3L7, at The Prince Charles Hospital (TPCH, Brisbane, Queensland, Australia) characterized by the *mexZ-*M3 and *lasR-*L7 alleles [9]. This strain infected 7.6% adults with CF in 2007–2009 and 6.4% adults with CF in 2011 [9]. Patients infected with the M3L7 sub-type had greater treatment requirements and a higher 3-year risk of death or lung transplantation compared to patients infected with other AUST-02 sub-types and *P*. *aeruginosa* strains [9].

It is widely recognized that there is substantial phenotypic and genetic intra-strain *P*. *aeruginosa* population diversity within the chronically infected CF airway with the emergence of multiple clonal lineages and strains with antibiotic resistance, hypermutation (caused by mutations in DNA mismatch repair [MMR] genes) and pathoadaptive mutations that enable adaptation to the CF airways all demonstrated [10–18].

Whilst the M3L7 sub-type has been associated with increased antibiotic resistance compared to other *P*. *aeruginosa* strains including other sub-types of AUST-02, the diversity within an individual is unknown [9]. Furthermore, antibiotic resistance mechanisms are recognized as diverse amongst *P*. *aeruginosa* but the within-host variation is less clear [19]. Here we used whole genome sequencing (WGS) to investigate the M3L7 sub-type isolated during and after intravenous antibiotic treatment of a pulmonary exacerbation with the principle aim of constructing a genomic analysis of antibiotic resistance gene mutations.

## Materials and methods

### Overview

This study focused on a single patient with CF identified as harboring the AUST-02 shared strain sub-type, M3L7 (hereafter referred to as M3L7), on the basis of *mexZ* and *lasR* DNA sequencing [9]. In brief, sputum samples were collected at five time-points during and after treatment of an acute pulmonary exacerbation (a 3 month period). All AUST-02 isolates identified were screened for the M3L7 sub-type. M3L7 isolates were then selected for WGS on the basis of antibiotic susceptibility data.

### Ethical approval

Ethics approval for this project was granted under HREC/13/QPCH/127 by TPCH Human and Research Ethics Committee, Metro North Hospital and Health Service, Brisbane, Queensland, Australia and the participant provided written, informed consent.

### Patient details

The patient was a 40-year old male with severe lung disease (forced expiratory volume in one second of 44% predicted), who was admitted to TPCH for treatment of an acute pulmonary exacerbation in February 2014. The patient previously tested M3L7 positive in 2007 (isolate ID, AUS951) [9].

**Fig 1:**
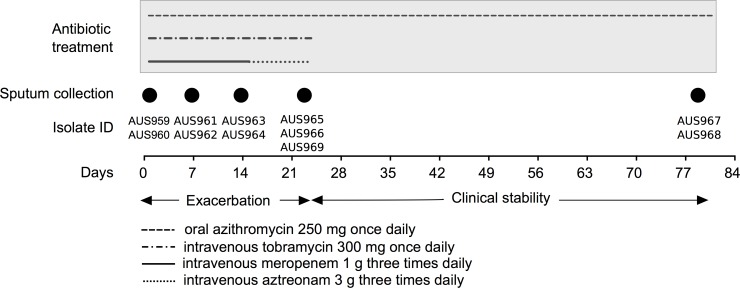
Timeline of antibiotic treatment and sputum collection. The length and timing of a specific antibiotic treatment is shown by different line-styles. The days of sputum collection are shown by filled-in circles (days 1, 7, 14, 23) and 8 weeks following discharge (day 79). Isolates cultured at each time-point and used in this study are also indicated.

Hypersensitivity reactions had previously complicated courses of extended activity anti-pseudomonal penicillin (ticarcillin/clavulanate) and cephalosporin antibiotics (ceftazidime) thus limiting the choice of β-lactam antibiotics to treat pulmonary exacerbations. Based on the patient’s previous response to treatment, meropenem (1 g three times daily) and tobramycin (300 mg once daily), which have different mechanisms of action, were administered intravenously on admission. However, on this occasion meropenem was ceased and substituted with intravenous aztreonam (3 g three times daily) on day 14 due to a suboptimal clinical response and was continued until hospital discharge (day 23). Clinical stability was maintained until outpatient follow-up 8 weeks later (from date of discharge). The timeline of antibiotic treatment is shown in Fig 1.

The patient received long-term oral azithromycin (250 mg once daily) throughout the study (Fig 1). The patient did not receive alternate month inhaled therapies (intolerance with nebulized colistin and patient declined nebulized tobramycin due to perceived lack of efficacy).

### Patient isolates

Expectorated sputum was collected (Fig 1) and processed to isolate single colonies (method described in S1 File). Presumptive *P*. *aeruginosa* isolates (*n* = 48) were randomly selected from each sputa collected at five time-points: day 1 (start of intravenous antibiotics), day 7 (week 1), day 14 (week 2), day 23 (completion of intravenous antibiotics) and outpatient follow-up (8-weeks later). All isolates underwent strain typing using an allele-specific AUST-02 PCR (S1 File) and Sequenom iPLEX SNP-based strain typing [20]. All confirmed AUST-02 isolates were further differentiated by *mexZ*-specific PCR and *lasR* gene sequencing as described previously [9]. Susceptibility testing to 11 anti-pseudomonal antibiotics (Table 1) was performed using the disc diffusion assay with isolates categorized as resistant, intermediate resistant or susceptible to each antibiotic according to Clinical and Laboratory Standards Institute (CLSI) breakpoint guidelines [21].

**Table 1:** *In vitro* antibiotic susceptibility profiles of AUST-02 isolates (*n* = 134).

Antibiotic	Susceptibility profile number*																		
	1	2	3	4	5	6	7	8	9	10	11	12	13	14	15	16	17	18	19
Ciprofloxacin	**S**	**R**	**I**	**R**	**S**	**I**	**S**	**S**	**I**	**R**	**S**	**I**	**I**	**I**	**I**	**R**	**I**	**R**	**I**
Tobramycin	**S**	**S**	**R**	**R**	**R**	**S**	**I**	**S**	**I**	**I**	**S**	**S**	**S**	**S**	**I**	**S**	**R**	**I**	**S**
Amikacin	R	R	R	R	R	R	R	I	R	R	R	S	I	R	R	S	R	R	R
Aztreonam	R	R	R	R	R	R	R	R	R	R	R	S	R	I	R	R	R	R	R
Ceftazidime	R	R	R	R	R	R	R	R	R	R	R	S	R	R	R	R	R	R	R
Cefepime	R	R	R	R	R	R	R	R	R	R	S	R	R	R	R	R	R	R	S
Imipenem	R	R	R	R	R	R	R	R	R	R	R	S	R	R	R	R	R	R	R
Meropenem	R	R	R	R	R	R	R	R	R	R	R	S	R	R	S	R	R	R	I
Ticarcillin/clavulanate	R	R	R	R	R	R	R	R	R	R	R	S	R	R	R	R	R	R	R
Colistin sulphate^II^	S	S	S	S	S	S	S	S	S	S	S	S	S	S	S	S	S	S	R
Polymyxin B^II^	S	S	S	S	S	S	S	S	S	S	S	S	S	S	S	S	R	R	R
Isolates at each time-point; no.																			
Day 1	28^‡^	1	3^‡^	2	0	3	3	1	2	1	0	0	0	0	1	0	0	0	1
Day 7	4	1	3^‡^	2	1	1	0	1	0	0	0	0	1	1^‡^	0	0	0	0	0
Day 14	0	10^‡^	5	10^‡^	6	2	1	0	0	0	0	0	0	0	0	1	1	1	0
Day 23	3^‡^	4^‡^	5	1^‡^	5	4	2	0	0	1	0	1	0	0	0	0	0	0	0
Outpatient follow-up	0	3	0	1	3	1^‡^	0	1	0	0	1^‡^	0	0	0	0	0	0	0	0
Total number of isolates; no. (%)	35	19	16	16	15	11	6	3	2	2	1	1	1	1	1	1	1	1	1
	(26)	(14)	(12)	(12)	(11)	(8)	(4)	(2)	(1.5)	(1.5)	(1)	(1)	(1)	(1)	(1)	(1)	(1)	(1)	(1)
Isolates selected for WGS	AUS960	AUS963	AUS959	AUS964		AUS968					AUS967			AUS962					
	AUS965	AUS969^†^	AUS961	AUS966															

*Breakpoints defined by the Clinical and Laboratory Standards Institute [21]

^†^The sequenced M3L43 isolate (isolate ID, AUS969) was derived from the M3L7 sub-type having acquired an additional SNP in the *lasR* gene (S1 Table)

^‡^Isolates selected for WGS from these time-points

^II^Chromosomally encoded genes associated with polymyxin resistance were not investigated as all isolates sequenced were classed as susceptible to colistin sulphate and polymyxin B; antibiotic susceptibilities in bold, greatest phenotypic diversity observed.

*Abbreviations*: R, resistant; I, intermediate resistant; S, susceptible; WGS, whole-genome sequencing.

### Selection of isolates for whole genome sequencing

Based on the disc diffusion susceptibility data, 11 isolates were selected for WGS (Table 1 and Fig 1). Two M3L7 isolates with a different susceptibility profile were chosen at each time-point (day 1, AUS959 and AUS960; day 7, AUS961 and AUS962; day 14, AUS963 and AUS964; day 23, AUS965 and AUS966; Follow-up, AUS967 and AUS968; Table 1). An additional isolate (AUS969; M3L43) that was cultured at day 23, harboring a different *lasR* sequence, also underwent WGS (Table 1).

### Whole-genome sequencing

Preparation of genomic DNA for WGS was undertaken using the UltraClean® Microbial DNA Isolation Kit (Mo Bio) with several modifications as follows: prior to DNA extraction, samples were placed on ice (1 hour); the pelleted sample was washed with 0.9% saline and centrifuged at 14,000 x g for 1 min; during cell lysis, the samples were heated to 70°C for 10 mins with bump vortexing for 15 s every 2 mins; during protein removal, the centrifugation speed used was 12,000 x g; during re-suspension, the DNA was eluted to a final volume of 100 μL. Quantity and quality of the genomic DNA preparations was determined using NanoDrop Spectrophotometry, Quant-iT™ PicoGreen® dsDNA Reagent chemistry, and 0.8% visual gel analysis. Library preparation (Truseq), qPCR (TapeStation, Agilent Genomics) and WGS using the Illumina HiSeq 2500 platform with 100 bp paired-end read chemistry were carried out at the Australian Genome Research Facility, Melbourne, Australia.

### Genome mapping and assembly

Reads were examined for contamination using Kraken (v0.10.4), quality filtered with Nesoni (v0.128), and mapped to the PAO1 reference genome (Accession number: NC_002516), using SHRiMP2, as implemented in Nesoni [22–25]. SNPs and small insertions or deletions were called using Nesoni. WGS reads were assembled with Velvetoptimiser (v2.2.5) and Velvet (v1.2.10) [26,27]. Resulting contigs were reordered against PAO1 using Mauve (v.2.4.0) and annotated using Prokka (v1.10) [28,29]. Gene annotations from PAO1 were used as the primary reference.

### Phylogenetic analysis

The core genome alignment was generated from the consensus sequence of the mapped reads against PAO1 comprising a total of 1753 polymorphic positions. Recombination filtering was carried out using Gubbins (v1.4.9), with 1534 polymorphic positions remaining after filtering [30].

Maximum-Likelihood phylogenetic trees were reconstructed from the pre-filtered and recombination filtered core genome alignment using RAxML (v8.1.15), with the rapid bootstrap option (-f a) and the general time reversible model with a gamma distribution of invariant sites [31]. The Lewis correction for ascertainment bias (–m ASC_GTRGAMMA–asc_corr lewis) was used for the recombination filtered SNP-only alignment. The genome of a different AUST-02 strain sub-type (M3L1; isolate ID, AUS970) identified as part of an ongoing study by our group from another CF patient was used as an outgroup to root the tree.

### Etest® susceptibility testing

In order to obtain minimum inhibitory concentrations (MICs) of the 11 anti-pseudomonal antibiotics (that were used to select isolates for WGS), each M3L7 isolate that underwent WGS was inoculated onto Mueller-Hinton Agar (Thermo Fisher Scientific) with susceptibility testing determined by Etest^®^ (BioMérieux) according to the manufacturer’s instructions. Breakpoints were categorized as susceptible, intermediate resistant or resistant as defined by the CLSI guidelines [21].

### Growth curve analysis

The fitness of the M3L7 sub-type was assessed by determining the total viable count of M3L7 to that of PAO1 over 24 hours and comparing the doubling time during the logarithmic phase of growth. The initial inoculum of each M3L7 isolate (*n* = 11) and PAO1 (*n* = 3, biological replicates) was prepared to ~1 X 10^5^ colony-forming units (CFU)/mL in Luria-Bertani Broth (20 mL). Bacterial cultures were incubated aerobically with shaking (200 rpm) at 37°C and total viable counts were enumerated on Mueller-Hinton agar at 0, 2, 4, 6, 10 and 24 hours.

### Identification of genes associated with antibiotic resistance, hypermutation and pathoadaptation

Screening for antimicrobial resistance genes was performed by a BLAST alignment of all predicted coding regions against CARD (Comprehensive Antimicrobial Resistance Database), MERGEM (Mobile Elements and Resistance Genes database Enhanced for Metagenomics) and ResFinder databases [32–34].

Single or multiple nucleotide substitutions, insertions or deletions in chromosomally encoded genes (*n* = 136) associated with antibiotic resistance, hypermutation and pathoadaptive genes (identified based on a literature search; S2 Table), and intergenic regions were determined using both read mapping and sequence alignment of assembled contigs. Variants were processed and visualized using Nesoni’s nway function and the Harvest suite of tools (Parsnp and Gingr) (v1.1.2) [35]. Non-synonymous mutations were manually inspected and only unambiguous SNP calls were included in the analysis. Functionally important mutations were defined as premature stop codons or frame-shift mutations and recognized point mutations within the quinolone resistance-determining regions (QRDRs) of *gyrA*, *gyrB*, *parC* and *parE* [19,36]. The effect of amino acid changes on protein function (effect or no effect) can be predicted using computational algorithms [37]. PROVEAN (Protein Variation Effect Analyzer) was used to filter the remaining missense and in-frame mutations into those that are predicted to be functionally important or neutral using the default score thresholds [38]. A ternary plot of amino acid variants was constructed using EvolView [39].

### Whole genome comparisons

Comparative genomic analyses were also performed to identify larger genomic variations using Parsnp, Gingr, BRIG (BLAST Ring Image Generator), Roary, ACT (Artemis Comparison Tool), bandage (a Bioinformatics Application for Navigating *De novo* Assembly Graphs Easily) and BLAST [35,40–43]. Prophage sequences were annotated with PHAST (PHAge Search Tool) [44].

### Accession numbers

Genome sequence data was deposited at the European Nucleotide Archive under study PRJEB14771 with accession identifiers ERS1245422 to ERS1245432. Samples AUS940, AUS951 and AUS970 are available as part of a separate study (PRJEB14781).

## Results and discussion

The *P*. *aeruginosa* AUST-02 strain sub-type M3L7 infects the airways of some patients with CF in Queensland and has been associated with antibiotic resistance [9]. We performed whole genome analyses of 11 M3L7 isolates isolated over a 3 month period, from a single patient and subsequently, investigated mutations in genes associated with antibiotic resistance, hypermutation and pathoadaptation.

In this study we demonstrate that the M3L7 population was heterogeneous when observed during treatment of a pulmonary exacerbation and subsequent recovery period highlighting the complexity of chronic M3L7 airway infection in CF. We detected variation in chromosomally encoded genes associated with antibiotic resistance, which may reflect adaptation of M3L7 isolates to different antibiotic selection pressures in spatially heterogeneous regions of the airway [14,45]. Moreover, our study design allowed us to identify mutations in target genes, shared by each isolate sequenced, that might also contribute to the high-level antibiotic resistance of the M3L7 sub-type.

Notably, despite the patient harboring multi-drug resistant M3L7 isolates, clinical response to the antibiotic regimen administered was achieved after a lengthy course of intravenous antibiotics (23 days). This corroborates with others who showed a lack of correlation between predicted susceptibility and clinical outcomes in CF [46–48].

### Phenotypic variation in ciprofloxacin and tobramycin resistance in the M3L7 population sampled

Genotyping confirmed 234/240 (97.5%) isolates as *P*. *aeruginosa* and showed that 134 (57%) belonged to the AUST-02 strain. Of these, 131 (98%) showed an M3L7 sub-type and three were of the M3L43 sub-type (2%). The M3L43 isolate (isolate ID, AUS969) was derived from the M3L7 sub-type having acquired an additional SNP in the *lasR* gene (S1 Table).

Disc diffusion susceptibility results for the 134 isolates are presented in Table 1. Studies elsewhere demonstrated that antibiotic susceptibility profiles might differ between isolates of the same strain, even when isolated from individual samples or different regions of the lung [13,45]. Such differences were noted here (19 different antibiotic susceptibility profiles observed; Table 1), with the greatest variation in antibiotic susceptibility detected for ciprofloxacin (resistant: *n* = 39/134, 29%; intermediate resistant: *n* = 35/134, 26%; susceptible: *n* = 60/134, 45%; Table 1) and tobramycin (resistant: *n* = 48/134, 36%; intermediate resistant: *n* = 12/134, 9%; susceptible: *n* = 74/134, 55%; Table 1). Between day 1 of treatment of a pulmonary exacerbation and at follow-up during clinical stability, resistance to ciprofloxacin and tobramycin also varied. Antibiotic resistance to both antibiotics within the *P*. *aeruginosa* population sampled was greatest at day 14 of treatment (ciprofloxacin resistant: *n* = 22/37, 59%; tobramycin resistant: *n* = 22/37, 59%; Table 1). Of interest, the patient was not prescribed ciprofloxacin during the 3 month assessment period; however, several courses of ciprofloxacin (oral, 750 mg twice daily for two weeks) were prescribed at an outpatient clinic in the prior 12 months, including one month prior to recruitment, for treatment of mild exacerbation episodes. Therefore, resistance may have been acquired previously in some isolates and maintained in the absence of ongoing ciprofloxacin exposure.

### M3L7 hypermutators and non-hypermutators co-exist in the CF airway

**Fig 2:**
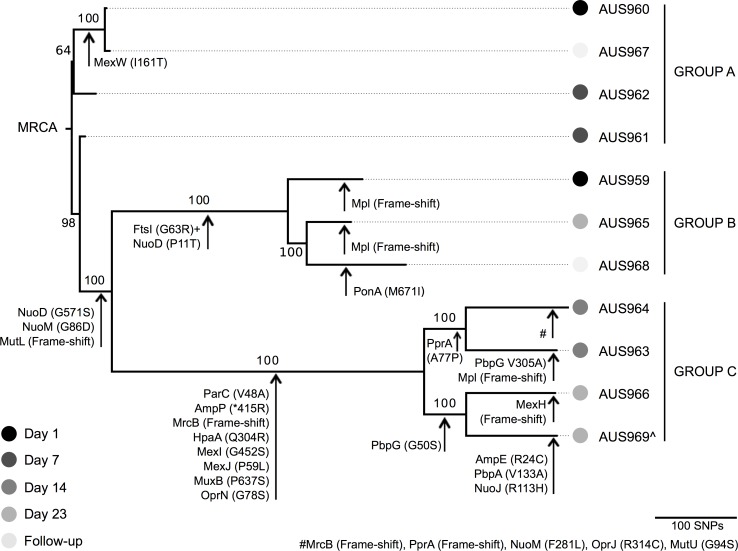
Phylogeny of M3L7 isolates from an individual with cystic fibrosis. Isolates were cultured from sputum samples during intravenous treatment of an acute pulmonary exacerbation (Days 1, 7, 14 and 23) and at outpatient follow-up 8 weeks later. The phylogenetic tree was constructed based on a core SNP alignment of 1753 nucleotides generated from read mapping against PAO1. The genome of a M3L1 strain (isolate ID, AUS970), sequenced as part of an ongoing study, was used as an outgroup to root the tree. Isolates in Groups B and C are hypermutators whilst those in Group A are non-hypermutators. The scale bar represents 100 nucleotide substitutions. Amino acid changes (compared to PAO1) in genes associated with antibiotic resistance and hypermutation are indicated using an arrow. *Premature stop codon; ^+^FtsI (G63C) identified in all other M3L7 isolates (Groups A and C); ^^^The M3L43 genotype (isolate ID, AUS969) is a derivative of the M3L7 sub-type; MRCA: most recent common ancestor. S2 Fig shows the phylogeny with predicted recombinant sites removed.

**Fig 3:**
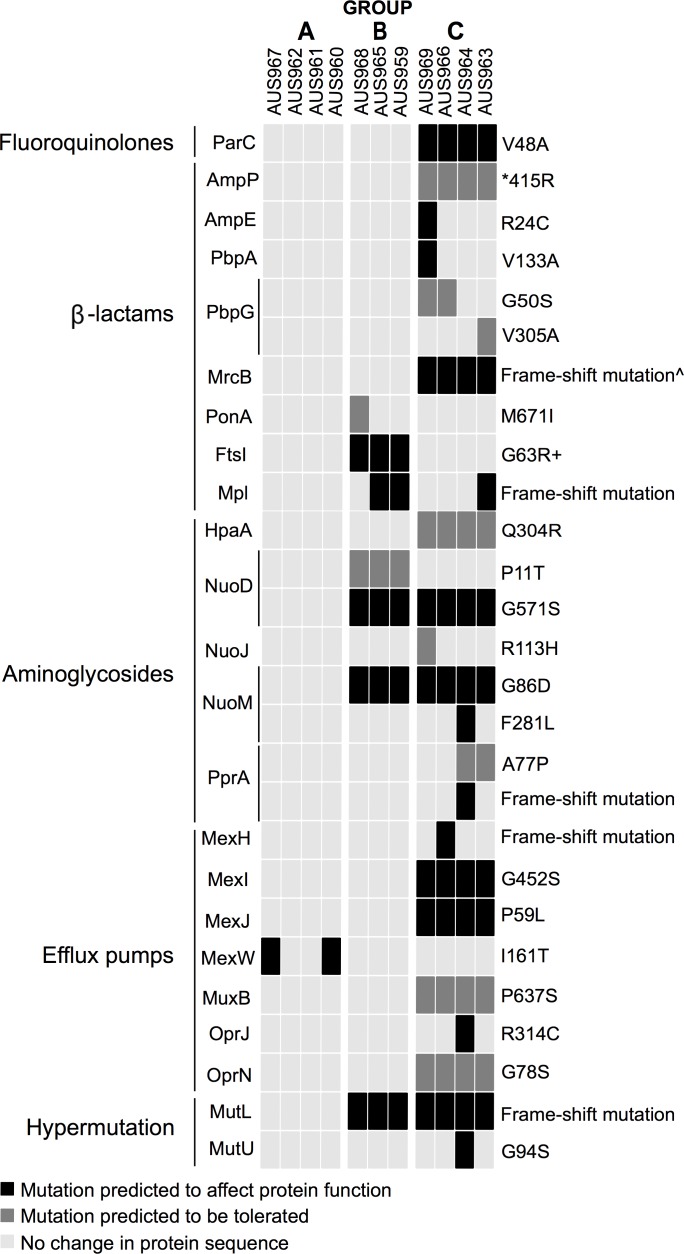
Ternary plot of amino acid sequence variation in genes associated with antibiotic resistance and hypermutation. M3L7 isolates were cultured from sputum samples during intravenous treatment of an acute pulmonary exacerbation (Days 1, 7, 14 and 23) and at outpatient follow-up 8 weeks later. Protein sequences (compared to PAO1) were grouped according to the resistance they confer or to their biological function and three colors are used to filter the proteins. The complete plot with the full set of chromosomally encoded proteins investigated, genetic mutations identified and PROVEAN scores are available in S1 Table. *Premature stop codon; ^+^FtsI (G63C) identified in all other M3L7 isolates (Group A and C); ^^^Additional frame-shift mutation detected in AUS964.

Our results corroborate with other recent studies, which have reported that multiple related *P*. *aeruginosa* lineages can co-exist within a single patient [10,12,15,16,45]. A previous study also reported the co-existence of hypermutators and non-hypermutators over nine years within a single patient with CF [17]. Although only 8% (*n* = 11/134) of the total AUST-02 population sampled over 3 months was sequenced, our data provide further evidence of this occurrence; the draft genomes of the M3L7 sub-type comprised 96–133 contigs with a total length between 6.19 to 6.24 Mbp (S1 Fig and S3 Table). Isolates could be stratified into 1 of 3 phylogenetic groups (Group A; Group B; Group C) as shown in Fig 2 and isolates within these distinct groups co-existed throughout the sampling period. Longer branch lengths were found to separate isolates within Groups B and C from Group A. The higher number of SNPs that were observed for isolates of Groups B and C is suggestive of a hypermutable phenotype that may be caused by mutations in MMR genes [16,49]. In fact, a frame-shift mutation caused by a 1 bp deletion (1660delT) within the MMR gene, *mutL*, was identified for Groups B and C, but not Group A, consistent with the acquisition of this mutation prior to the divergence of Groups B and C (Figs 2 and 3).

Of note, the M3L7 isolate (isolate ID, AUS951) cultured from the patient in 2007 clustered with Group A non-hypermutator isolates and was most closely related to AUS961 (differed by 14 SNPs, data not shown).

### Variation in chromosomally encoded genes was observed

Based on the CLSI guidelines, all isolates were categorized as resistant (S3 Fig) to aztreonam (MIC range 64 to >256 mg/L), ceftazidime (MIC >256 mg/L), cefepime (MIC >256 mg/L), imipenem (MIC >32 mg/L), meropenem (MIC >32 mg/L) and ticarcillin/clavulanate (MIC >256 mg/L) and resistant or intermediate resistant to amikacin (range MIC 32 to >256 mg/L). In contrast, all isolates were categorized as susceptible (S3 Fig) to colistin (MIC range 0.125–0.5 mg/L) and polymyxin B (MIC range 0.25–1 mg/L). Ciprofloxacin (MIC range 0.5–8 mg/L) and tobramycin (MIC range 2–64 mg/L) showed the greatest variability in MICs (S3 Fig) with isolates categorized as resistant, intermediate resistant and susceptible (similar to disc diffusion results that were used to select isolates for WGS, Table 1). The S2 File contains the data set of the individual isolate MICs.

Analysis of each M3L7 genome via multiple antibiotic resistance gene databases failed to identify acquired antibiotic resistance genes suggesting M3L7 resistance is principally driven by chromosomal mutations. In total, mutations, shared by all 11 isolates, were identified in 49 chromosomal genes previously implicated in conferring *P*. *aeruginosa* antibiotic resistance (S1 Table). Whilst the impact of most SNPs is unknown, functionally important mutations are likely to include frame-shift mutations (*mexA*, *mexT*) and premature stop codons (*oprD*, *mexB*). Shared mutations that have been previously described were also identified including premature stop codons within *oprD* (E176* identified in M3L7) that could result in loss of the outer membrane porin and contribute to carbapenem resistance and an 8 bp deletion (226_233delCGGCCAGC) in *mexT* characteristic of *nfxC*-mutants that display *mexEF-oprN* overexpression but reduced *oprD* transcription [19,50–53]. A point mutation in *ampC* (T105A) that confers reduced susceptibility to imipenem, ceftazidime and cefepime when the enzyme is overexpressed was also identified [54]. Although no recognized mutations were observed that easily explain *ampC* derepression, a novel nucleotide substitution within the *ampC-ampR* intergenic region (PAO1, TAGAAT; M3L7, TATATT) was identified in all 11 isolates and was found to reside within the -10 promoter region of *ampC*, which could affect production of AmpC and anti-pseudomonal penicillin and cephalosporin resistance [55].

WGS revealed small genetic variations in 21 genes that may impact the activity of β-lactams (e.g. mutations in penicillin-binding proteins), aminoglycosides (e.g. mutations that may affect uptake) and multi-drug efflux pumps (e.g. mutations in cytoplasmic membrane components) between M3L7 groups or individual isolates (Figs 2 and 3).

There were no mutations that could be easily correlated with ciprofloxacin and tobramycin MIC variability. A novel point mutation outside the QRDR was observed in *parC* (V48A) amongst Group C isolates (3/4 of these isolates were resistant to ciprofloxacin [4–8 mg/L] and the remaining isolate was intermediate resistant [2 mg/L]; Fig 2), which was acquired by this group after divergence from Group B and was predicted *in silico* to affect protein function (Fig 3). Other mutations identified that potentially contribute to ciprofloxacin resistance (e.g. *gyrA*, T83I; *parE*, A473V; *mexT*, 8 bp deletion) were shared by all isolates [MIC range, 0.5–8 mg/L] (S1 Table) [36,51]. Mutations only acquired by the tobramycin-resistant isolate (AUS961, 64 mg/L) and associated with aminoglycoside resistance were not identified and isolates with intermediate resistance to amikacin (AUS963, 32 mg/L) and tobramycin (AUS966, 12 mg/L) appeared genetically similar to isolates with resistant phenotypes when based on the panel of target chromosomally encoded genes investigated (Fig 2). Further work is required to investigate the effect of the mutations detected on antibiotic resistance and to determine their prevalence in a larger number of isolates [56].

It has been proposed that certain genes are involved in the parallel evolution and adaptation of different *P*. *aeruginosa* strains to the host [18]. Overall, 38/52 (73%) candidate pathoadaptive genes harbored small genetic variations in the M3L7 isolates sequenced (S1 Table) [18]. Some mutations found in these pathoadaptive genes (S1 Table) were acquired after divergence of groups (e.g. a frame-shift mutation in *mucA* was acquired by Group B isolates) [18]. Furthermore, growth of the M3L7 sub-type (mean doubling time, 59 mins) was slower than PAO1 (mean doubling time, 32 mins) suggesting that an *in vitro* fitness cost is associated with the sub-type (S4 Fig). The S2 File contains the data set of the total viable counts for each isolate.

Altogether the data emphasize the complexity of chronic M3L7 infection where genotypically diverse sub-lineages, with variation in antibiotic resistance and pathoadaption genes, co-exist.

### The patient was infected with multiple *Pseudomonas aeruginosa* strains

Recently there has been a substantial increase in the survival of people with CF, even in those with advanced pulmonary disease [57], and consequently the lifetime exposure to antibiotics is rising. In parallel, the emergence of antibiotic-resistant CF pathogens are increasingly common and antibiotic toxicity challenges the effectiveness and limits the choice of antibiotic regimens, as described for the patient included here. In addition to infection with M3L7, the patient was co-infected with two other recognized shared strain-types, AUST-06 (*n* = 25/234; 11%) and AUST-07 (*n* = 75/234; 32%) that have been found in a number of patients attending TPCH Adult CF Centre [2]. This further demonstrates the challenges of treating chronic *P*. *aeruginosa* infection where multiple strain types and strain sub-lineages with differing susceptibility profiles may all be present [10,12,15,45].

Therefore, there is an urgent need for the development of new antibiotics to treat multi-drug resistant *P*. *aeruginosa*, new antibiotic formulations and adjuvant antimicrobial agents that can improve the action of current treatments [58–60]. In the future it may be possible to commence a personalized treatment approach that will consider the resistance profile of the complex *P*. *aeruginosa* population and limit pathoadaptation of bacteria residing within the lungs.

### Limitations

A potential limitation of this study is the relatively small proportion of available isolates that we have sequenced. Further work is required to determine the prevalence of each M3L7 group (A, B, C) at different time-points or determine if one group dominated during or after treatment of an acute pulmonary exacerbation and if there is evidence of periodic selection [10]. Given the small sample size, we also could not directly correlate susceptibility profile and genotype but the results indicate that discrepancies are likely and that a cautious approach should be taken if susceptibility is predicted using genomic analyses of single isolates in chronic infections given the diversity observed [56].

## Conclusions

We have provided a short-term within-host analysis of an antibiotic resistant *P*. *aeruginosa* strain sub-type in CF using whole genome data. The findings suggest that diversity exists in the M3L7 population and variation in chromosomally encoded genes associated with resistance, hypermutation and pathoadaptation were observed. Discrete mutational events might have accumulated to enable the M3L7 sub-type to continually evolve, diverge and adapt to the spatially heterogeneous niches of the CF airways. These results have broader implications for interpreting antimicrobial susceptibility profiles predicted by genomic analyses of individual isolates or metagenomic analysis of sputum samples during acute pulmonary exacerbations.

## Supporting information

S1 Fig Complete genome sequences of the M3L7 isolates (listed in the key) compared to the AUS940 reference genome (M3L7 isolate from a different patient identified from an ongoing study) using BLAST Ring Image Generator (BRIG) software. Each genome is grouped according to phylogeny. *Two strains (Group A, AUS961 and AUS962) have lost a 40 Kbp prophage between 3900 and 4000 Kbps. This prophage was not annotated with antibiotic resistance genes.
(TIF)

S2 Fig Phylogeny of M3L7 isolates following recombination filtering. Recombination filtering was carried out using Gubbins. The phylogenetic tree was constructed based on a core SNP alignment of 1534 nucleotides generated from read mapping against PAO1. The sequenced genome of a M3L1 strain (isolate ID, AUS970), as part of an ongoing study, was used as an out-group to root the tree. The scale bar represents 100 nucleotide substitutions. **Indicates branches with 100% support from 1000 bootstrap replicates. *Abbreviation*: MRCA, most recent common ancestor.
(TIF)

S3 Fig Minimum inhibitory concentrations (mg/L) of M3L7 isolates (*n* = 11) against 11 anti-pseudomonal antibiotics. Isolates were categorized as resistant, intermediate or susceptible according to CLSI guidelines. The median and interquartile range are shown. Any isolates recorded as having a MIC greater than the maximum (>256 mg/L or >32 mg/L) value on the Etest^®^ strip are shown as double the maximum concentration.
(PDF)

S4 Fig Geometric mean (with 95% confidence intervals) total viable counts expressed as colony-forming units (CFU)/mL of the M3L7 sub-type (*n* = 11) compared to PAO1 (*n* = 3) grown in Luria-Bertani Broth. (PDF)

S1 Table Ternary plot of amino acid sequence variation. (XLSX)

S2 Table Literature search. (DOCX)

S3 Table General genome features of isolates within the M3L7 sub-type. (DOCX)

S1 File Collection and processing of sputum samples and Allele-specific PCR for AUST-02. (DOCX)

S2 File Data sets of individual data points relating to S3 Fig and S4 Fig. (DOCX)

## References

[1] Cystic Fibrosis Trust. UK Cystic Fibrosis Registry 2014 Annual Report. 2015.

[2] Kidd TJ, Ramsay KA, Hu H, Marks GB, Wainwright CE, Bye PT, et al Shared *Pseudomonas aeruginosa* genotypes are common in Australian cystic fibrosis centres. Eur Respir J. 2013;41(5):1091–100. 10.1183/09031936.00060512 22878877

[3] Jones AM, Dodd ME, Doherty CJ, Govan JR, Webb AK. Increased treatment requirements of patients with cystic fibrosis who harbour a highly transmissible strain of *Pseudomonas aeruginosa*. Thorax. 2002;57(11):924–5. 10.1136/thorax.57.11.924 12403871 PMC1746227

[4] Aaron SD, Vandemheen KL, Ramotar K, Giesbrecht-Lewis T, Tullis E, Freitag A, et al Infection with transmissible strains of *Pseudomonas aeruginosa* and clinical outcomes in adults with cystic fibrosis. JAMA. 2010;304(19):2145–53. 10.1001/jama.2010.1665 21081727

[5] Fothergill JL, Walshaw MJ, Winstanley C. Transmissible strains of *Pseudomonas aeruginosa* in cystic fibrosis lung infections. Eur Respir J. 2012;40(1):227–38. 10.1183/09031936.00204411 22323572

[6] Kidd TJ, Soares Magalhaes RJ, Paynter S, Bell SC, Group ACI. The social network of cystic fibrosis centre care and shared *Pseudomonas aeruginosa* strain infection: a cross-sectional analysis. Lancet Respir Med. 2015;3(8):640–50. 10.1016/S2213-2600(15)00228-3 26208994

[7] Robinson P, Carzino R, Armstrong D, Olinsky A. Pseudomonas cross-infection from cystic fibrosis patients to non-cystic fibrosis patients: implications for inpatient care of respiratory patients. J Clin Microbiol. 2003;41(12):5741 10.1128/JCM.41.12.5741.2003 14662972 PMC309033

[8] O'Carroll MR, Syrmis MW, Wainwright CE, Greer RM, Mitchell P, Coulter C, et al Clonal strains of *Pseudomonas aeruginosa* in paediatric and adult cystic fibrosis units. Eur Respir J. 2004;24(1):101–6. 15293611 10.1183/09031936.04.00122903

[9] Tai AS, Bell SC, Kidd TJ, Trembizki E, Buckley C, Ramsay KA, et al Genotypic Diversity within a Single *Pseudomonas aeruginosa* Strain Commonly Shared by Australian Patients with Cystic Fibrosis. PLoS One. 2015;10(12):e0144022 10.1371/journal.pone.0144022 26633539 PMC4669131

[10] Diaz Caballero J, Clark ST, Coburn B, Zhang Y, Wang PW, Donaldson SL, et al Selective Sweeps and Parallel Pathoadaptation Drive *Pseudomonas aeruginosa* Evolution in the Cystic Fibrosis Lung. MBio. 2015;6(5):e00981–15. 10.1128/mBio.00981-15 26330513 PMC4556809

[11] Smith EE, Buckley DG, Wu Z, Saenphimmachak C, Hoffman LR, D'Argenio DA, et al Genetic adaptation by *Pseudomonas aeruginosa* to the airways of cystic fibrosis patients. Proc Natl Acad Sci U S A. 2006;103(22):8487–92. 10.1073/pnas.0602138103 16687478 PMC1482519

[12] Chung JC, Becq J, Fraser L, Schulz-Trieglaff O, Bond NJ, Foweraker J, et al Genomic variation among contemporary *Pseudomonas aeruginosa* isolates from chronically infected cystic fibrosis patients. J Bacteriol. 2012;194(18):4857–66. 10.1128/JB.01050-12 22753054 PMC3430303

[13] Mowat E, Paterson S, Fothergill JL, Wright EA, Ledson MJ, Walshaw MJ, et al *Pseudomonas aeruginosa* population diversity and turnover in cystic fibrosis chronic infections. Am J Respir Crit Care Med. 2011;183(12):1674–9. 10.1164/rccm.201009-1430OC 21297072

[14] Dettman JR, Rodrigue N, Aaron SD, Kassen R. Evolutionary genomics of epidemic and nonepidemic strains of *Pseudomonas aeruginosa*. Proc Natl Acad Sci U S A. 2013;110(52):21065–70. 10.1073/pnas.1307862110 24324153 PMC3876195

[15] Williams D, Evans B, Haldenby S, Walshaw MJ, Brockhurst MA, Winstanley C, et al Divergent, coexisting *Pseudomonas aeruginosa* lineages in chronic cystic fibrosis lung infections. Am J Respir Crit Care Med. 2015;191(7):775–85. 10.1164/rccm.201409-1646OC 25590983 PMC4407486

[16] Feliziani S, Marvig RL, Lujan AM, Moyano AJ, Di Rienzo JA, Krogh Johansen H, et al Coexistence and within-host evolution of diversified lineages of hypermutable *Pseudomonas aeruginosa* in long-term cystic fibrosis infections. PLoS Genet. 2014;10(10):e1004651 10.1371/journal.pgen.1004651 25330091 PMC4199492

[17] Marvig RL, Johansen HK, Molin S, Jelsbak L. Genome analysis of a transmissible lineage of *Pseudomonas aeruginosa* reveals pathoadaptive mutations and distinct evolutionary paths of hypermutators. PLoS Genet. 2013;9(9):e1003741 10.1371/journal.pgen.1003741 24039595 PMC3764201

[18] Marvig RL, Sommer LM, Molin S, Johansen HK. Convergent evolution and adaptation of *Pseudomonas aeruginosa* within patients with cystic fibrosis. Nat Genet. 2015;47(1):57–64. 10.1038/ng.3148 25401299

[19] Kos VN, Deraspe M, McLaughlin RE, Whiteaker JD, Roy PH, Alm RA, et al The resistome of *Pseudomonas aeruginosa* in relationship to phenotypic susceptibility. Antimicrob Agents Chemother. 2015;59(1):427–36. 10.1128/AAC.03954-14 25367914 PMC4291382

[20] Syrmis MW, Kidd TJ, Moser RJ, Ramsay KA, Gibson KM, Anuj S, et al A comparison of two informative SNP-based strategies for typing *Pseudomonas aeruginosa* isolates from patients with cystic fibrosis. BMC Infect Dis. 2014;14:307 10.1186/1471-2334-14-307 24902856 PMC4053291

[21] Clinical and Laboratory Standards Institute. Performance Standards for Antimicrobial Susceptibility Testing: Twenty-third Informational Supplement M100-S23. CLSI, Wayne, PA, USA, 2013.

[22] Wood DE, Salzberg SL. Kraken: ultrafast metagenomic sequence classification using exact alignments. Genome Biol. 2014;15(3):R46 10.1186/gb-2014-15-3-r46 24580807 PMC4053813

[23] https://github.com/Victorian-Bioinformatics-Consortium/nesoni.

[24] Stover CK, Pham XQ, Erwin AL, Mizoguchi SD, Warrener P, Hickey MJ, et al Complete genome sequence of *Pseudomonas aeruginosa* PAO1, an opportunistic pathogen. Nature. 2000;406(6799):959–64. 10.1038/35023079 10984043

[25] David M, Dzamba M, Lister D, Ilie L, Brudno M. SHRiMP2: sensitive yet practical SHort Read Mapping. Bioinformatics. 2011;27(7):1011–2. 10.1093/bioinformatics/btr046 21278192

[26] https://github.com/tseemann/VelvetOptimiser.

[27] Zerbino DR, Birney E. Velvet: algorithms for *de novo* short read assembly using de Bruijn graphs. Genome Res. 2008;18(5):821–9. 10.1101/gr.074492.107 18349386 PMC2336801

[28] Darling AE, Mau B, Perna NT. progressiveMauve: multiple genome alignment with gene gain, loss and rearrangement. PLoS One. 2010;5(6):e11147 10.1371/journal.pone.0011147 20593022 PMC2892488

[29] Seemann T. Prokka: rapid prokaryotic genome annotation. Bioinformatics. 2014;30(14):2068–9. 10.1093/bioinformatics/btu153 24642063

[30] Croucher NJ, Page AJ, Connor TR, Delaney AJ, Keane JA, Bentley SD, et al Rapid phylogenetic analysis of large samples of recombinant bacterial whole genome sequences using Gubbins. Nucleic Acids Res. 2015;43(3):e15 10.1093/nar/gku1196 25414349 PMC4330336

[31] Stamatakis A. RAxML version 8: a tool for phylogenetic analysis and post-analysis of large phylogenies. Bioinformatics. 2014;30(9):1312–3. 10.1093/bioinformatics/btu033 24451623 PMC3998144

[32] McArthur AG, Waglechner N, Nizam F, Yan A, Azad MA, Baylay AJ, et al The comprehensive antibiotic resistance database. Antimicrob Agents Chemother. 2013;57(7):3348–57. 10.1128/AAC.00419-13 23650175 PMC3697360

[33] Zankari E, Hasman H, Cosentino S, Vestergaard M, Rasmussen S, Lund O, et al Identification of acquired antimicrobial resistance genes. J Antimicrob Chemother. 2012;67(11):2640–4. 10.1093/jac/dks261 22782487 PMC3468078

[34] http://www.mergem.genome.ulaval.ca/.

[35] Treangen TJ, Ondov BD, Koren S, Phillippy AM. The Harvest suite for rapid core-genome alignment and visualization of thousands of intraspecific microbial genomes. Genome Biol. 2014;15(11):524 10.1186/s13059-014-0524-x 25410596 PMC4262987

[36] Bruchmann S, Dotsch A, Nouri B, Chaberny IF, Haussler S. Quantitative contributions of target alteration and decreased drug accumulation to *Pseudomonas aeruginosa* fluoroquinolone resistance. Antimicrob Agents Chemother. 2013;57(3):1361–8. 10.1128/AAC.01581-12 23274661 PMC3591863

[37] Prickett MH, Hauser AR, McColley SA, Cullina J, Potter E, Powers C, et al Aminoglycoside resistance of *Pseudomonas aeruginosa* in cystic fibrosis results from convergent evolution in the *mexZ* gene. Thorax. 2017;72(1):40–7. 10.1136/thoraxjnl-2015-208027 27325751 PMC5497499

[38] Choi Y, Chan AP. PROVEAN web server: a tool to predict the functional effect of amino acid substitutions and indels. Bioinformatics. 2015;31(16):2745–7. 10.1093/bioinformatics/btv195 25851949 PMC4528627

[39] Zhang H, Gao S, Lercher MJ, Hu S, Chen WH. EvolView, an online tool for visualizing, annotating and managing phylogenetic trees. Nucleic Acids Res. 2012;40(Web Server issue):W569–72. 10.1093/nar/gks576 22695796 PMC3394307

[40] Alikhan NF, Petty NK, Ben Zakour NL, Beatson SA. BLAST Ring Image Generator (BRIG): simple prokaryote genome comparisons. BMC Genomics. 2011;12:402 10.1186/1471-2164-12-402 21824423 PMC3163573

[41] Page AJ, Cummins CA, Hunt M, Wong VK, Reuter S, Holden MT, et al Roary: rapid large-scale prokaryote pan genome analysis. Bioinformatics. 2015;31(22):3691–3. 10.1093/bioinformatics/btv421 26198102 PMC4817141

[42] Carver TJ, Rutherford KM, Berriman M, Rajandream MA, Barrell BG, Parkhill J. ACT: the Artemis Comparison Tool. Bioinformatics. 2005;21(16):3422–3. 10.1093/bioinformatics/bti553 15976072

[43] Wick RR, Schultz MB, Zobel J, Holt KE. Bandage: interactive visualization of *de novo* genome assemblies. Bioinformatics. 2015;31(20):3350–2. 10.1093/bioinformatics/btv383 26099265 PMC4595904

[44] Zhou Y, Liang Y, Lynch KH, Dennis JJ, Wishart DS. PHAST: a fast phage search tool. Nucleic Acids Res. 2011;39(Web Server issue):W347–52. 10.1093/nar/gkr485 21672955 PMC3125810

[45] Jorth P, Staudinger BJ, Wu X, Hisert KB, Hayden H, Garudathri J, et al Regional Isolation Drives Bacterial Diversification within Cystic Fibrosis Lungs. Cell Host Microbe. 2015;18(3):307–19. 10.1016/j.chom.2015.07.006 26299432 PMC4589543

[46] Parkins MD, Rendall JC, Elborn JS. Incidence and risk factors for pulmonary exacerbation treatment failures in patients with cystic fibrosis chronically infected with *Pseudomonas aeruginosa*. Chest. 2012;141(2):485–93. 10.1378/chest.11-0917 21835906

[47] Hurley MN, Ariff AH, Bertenshaw C, Bhatt J, Smyth AR. Results of antibiotic susceptibility testing do not influence clinical outcome in children with cystic fibrosis. J Cyst Fibros. 2012;11(4):288–92. 10.1016/j.jcf.2012.02.006 22436723 PMC3382712

[48] Smith AL, Fiel SB, Mayer-Hamblett N, Ramsey B, Burns JL. Susceptibility testing of *Pseudomonas aeruginosa* isolates and clinical response to parenteral antibiotic administration: lack of association in cystic fibrosis. Chest. 2003;123(5):1495–502. 12740266 10.1378/chest.123.5.1495

[49] Oliver A, Canton R, Campo P, Baquero F, Blazquez J. High frequency of hypermutable *Pseudomonas aeruginosa* in cystic fibrosis lung infection. Science. 2000;288(5469):1251–4. 10818002 10.1126/science.288.5469.1251

[50] Kohler T, Epp SF, Curty LK, Pechere JC. Characterization of MexT, the regulator of the MexE-MexF-OprN multidrug efflux system of *Pseudomonas aeruginosa*. J Bacteriol. 1999;181(20):6300–5. 10515918 10.1128/jb.181.20.6300-6305.1999 PMC103763

[51] Maseda H, Saito K, Nakajima A, Nakae T. Variation of the *mexT* gene, a regulator of the MexEF-oprN efflux pump expression in wild-type strains of *Pseudomonas aeruginosa*. FEMS Microbiol Lett. 2000;192(1):107–12. 11040437 10.1111/j.1574-6968.2000.tb09367.x

[52] Sanbongi Y, Shimizu A, Suzuki T, Nagaso H, Ida T, Maebashi K, et al Classification of OprD sequence and correlation with antimicrobial activity of carbapenem agents in *Pseudomonas aeruginosa* clinical isolates collected in Japan. Microbiol Immunol. 2009;53(7):361–7. 10.1111/j.1348-0421.2009.00137.x 19563394

[53] Wolter DJ, Acquazzino D, Goering RV, Sammut P, Khalaf N, Hanson ND. Emergence of carbapenem resistance in *Pseudomonas aeruginosa* isolates from a patient with cystic fibrosis in the absence of carbapenem therapy. Clin Infect Dis. 2008;46(12):e137–41. 10.1086/588484 18462098

[54] Rodriguez-Martinez JM, Poirel L, Nordmann P. Extended-spectrum cephalosporinases in *Pseudomonas aeruginosa*. Antimicrob Agents Chemother. 2009;53(5):1766–71. 10.1128/AAC.01410-08 19258272 PMC2681535

[55] Caille O, Zincke D, Merighi M, Balasubramanian D, Kumari H, Kong KF, et al Structural and functional characterization of *Pseudomonas aeruginosa* global regulator AmpR. J Bacteriol. 2014;196(22):3890–902. 10.1128/JB.01997-14 25182487 PMC4248820

[56] Piddock LJ. Assess drug-resistance phenotypes, not just genotypes. Nat Microbiol. 2016;1(8):16120 10.1038/nmicrobiol.2016.120 27573119

[57] George PM, Banya W, Pareek N, Bilton D, Cullinan P, Hodson ME, et al Improved survival at low lung function in cystic fibrosis: cohort study from 1990 to 2007. BMJ. 2011;342:d1008 10.1136/bmj.d1008 21357627 PMC3045791

[58] Dassner AM, Sutherland C, Girotto J, Nicolau DP. *In vitro* activity of ceftolozane/tazobactam alone or with an aminoglycoside against multi-drug-resistant *Pseudomonas aeruginosa* from pediatric cystic fibrosis patients. Infect Dis Ther. 2016 [Epub ahead of print]. 10.1007/s40121-016-0141-y PMC5336416 27943223

[59] Campbell CT, McCaleb R, Manasco KB. New inhaled antimicrobial formulations for use in the cystic fibrosis patient population. Ann Pharmacother. 2016; 50(2):133–40. 10.1177/1060028015621916 26692274

[60] Christensen LD, van Gennip M, Jakobsen TH, Alhede M, Hougen HP, Høiby N, et al Synergistic antibacterial efficacy of early combination treatment with tobramycin and quorum-sensing inhibitors against *Pseudomonas aeruginosa* in an intraperitoneal foreign-body infection mouse model. J Antimicrob Chemother. 2012;67(5):1198–206. 10.1093/jac/dks002 22302561

