# Correction: Within-host whole genome analysis of an antibiotic resistant *Pseudomonas aeruginosa* strain sub-type in cystic fibrosis

**DOI:** 10.1371/journal.pone.0210929

**Published:** 2019-01-14

**Authors:** Laura J. Sherrard, Anna S. Tai, Bryan A. Wee, Kay A. Ramsay, Timothy J. Kidd, Nouri L. Ben Zakour, David M. Whiley, Scott A. Beatson, Scott C. Bell

PoxB (PA5297) has not been associated with antibiotic resistance (specifically beta-lactamase activity) as indicated in the original article's Supplementary Tables. The authors have provided updated and corrected S1 and S2 Tables here.

There is an error in the seventh sentence of the abstract. The correct sentence is: Small genetic variations, shared by all 11 isolates, were found in 48 genes associated with antibiotic resistance including frame-shift mutations (*mexA*, *mexT*), premature stop codons (*oprD*, *mexB*) and mutations in quinolone-resistance determining regions (*gyrA*, *parE*).

In the Materials and methods, there is an error in the first sentence of the second paragraph in the Identification of genes associated with antibiotic resistance, hypermutation and pathoadaptation section. The correct sentence is: Single or multiple nucleotide substitutions, insertions or deletions in chromosomally encoded genes (n = 135) associated with antibiotic resistance, hypermutation and pathoadaptive genes (identified based on a literature search; S2 Table), and intergenic regions were determined using both read mapping and sequence alignment of assembled contigs.

In the Results and discussion, there is an error in the second sentence of the second paragraph in the Variation in chromosomally encoded genes was observed section. The correct sentence is: In total, mutations, shared by all 11 isolates, were identified in 48 chromosomal genes previously implicated in conferring P. aeruginosa antibiotic resistance (S1 Table).

## Supporting information

S1 Table Ternary plot of amino acid sequence variation. (XLSX)

S2 Table Literature search. (DOCX)
