# Supplementary figures and images for: Within-host whole genome analysis of an antibiotic resistant Pseudomonas aeruginosa strain sub-type in cystic fibrosis
Source: PLoS One. 2017 Mar 8;12(3):e0172179. doi: 10.1371/journal.pone.0172179 (PMC5342179; doi:10.1371/journal.pone.0172179)

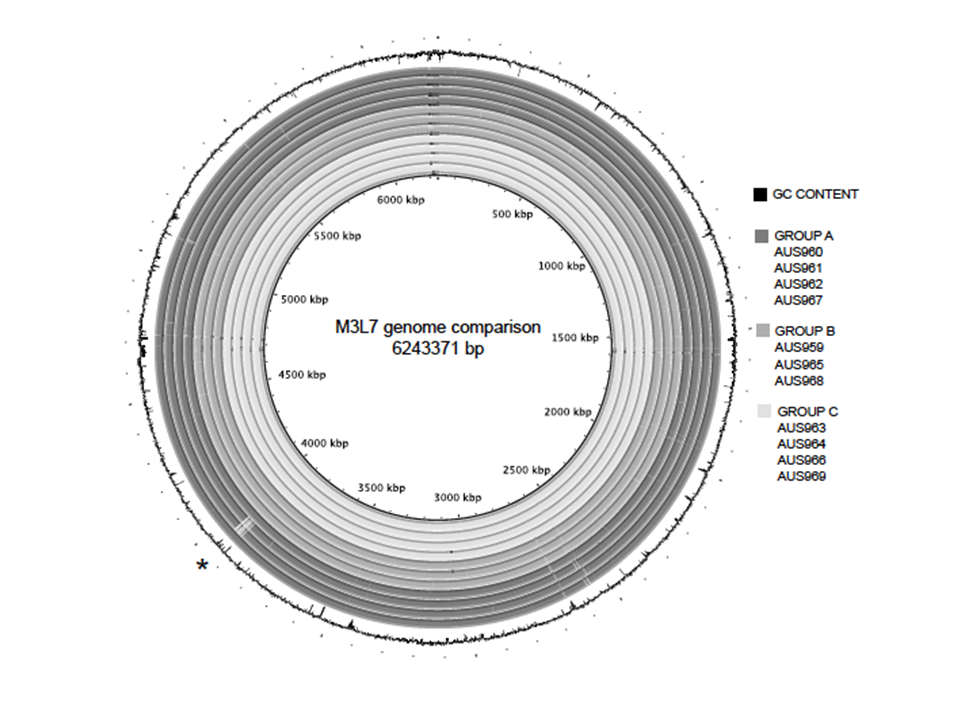

Supplement: S1 Fig — Each genome is grouped according to phylogeny. *Two strains (Group A, AUS961 and AUS962) have lost a 40 Kbp prophage between 3900 and 4000 Kbps. This prophage was not annotated with antibiotic resistance genes. (TIF) [file pone.0172179.s001.tif]

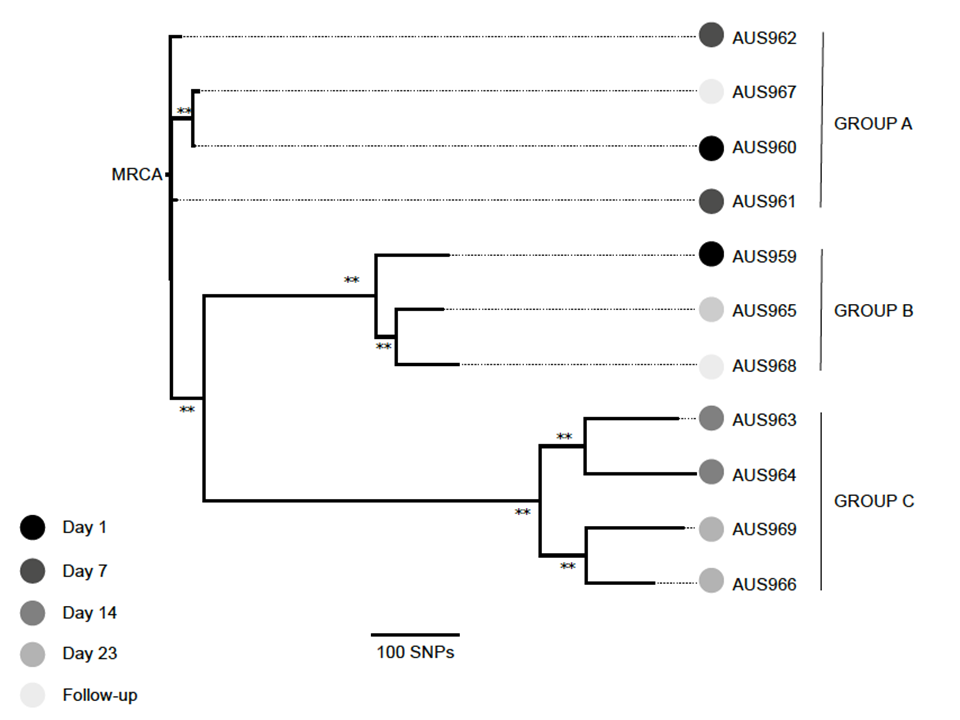

Supplement: S2 Fig — Recombination filtering was carried out using Gubbins. The phylogenetic tree was constructed based on a core SNP alignment of 1534 nucleotides generated from read mapping against PAO1. The sequenced genome of a M3L1 strain (isolate ID, AUS970), as part of an ongoing study, was used as an out-group to root the tree. The scale bar represents 100 nucleotide substitutions. **Indicates branches with 100% support from 1000 bootstrap replicates. Abbreviation: MRCA, most recent common ancestor. (TIF) [file pone.0172179.s002.tif]

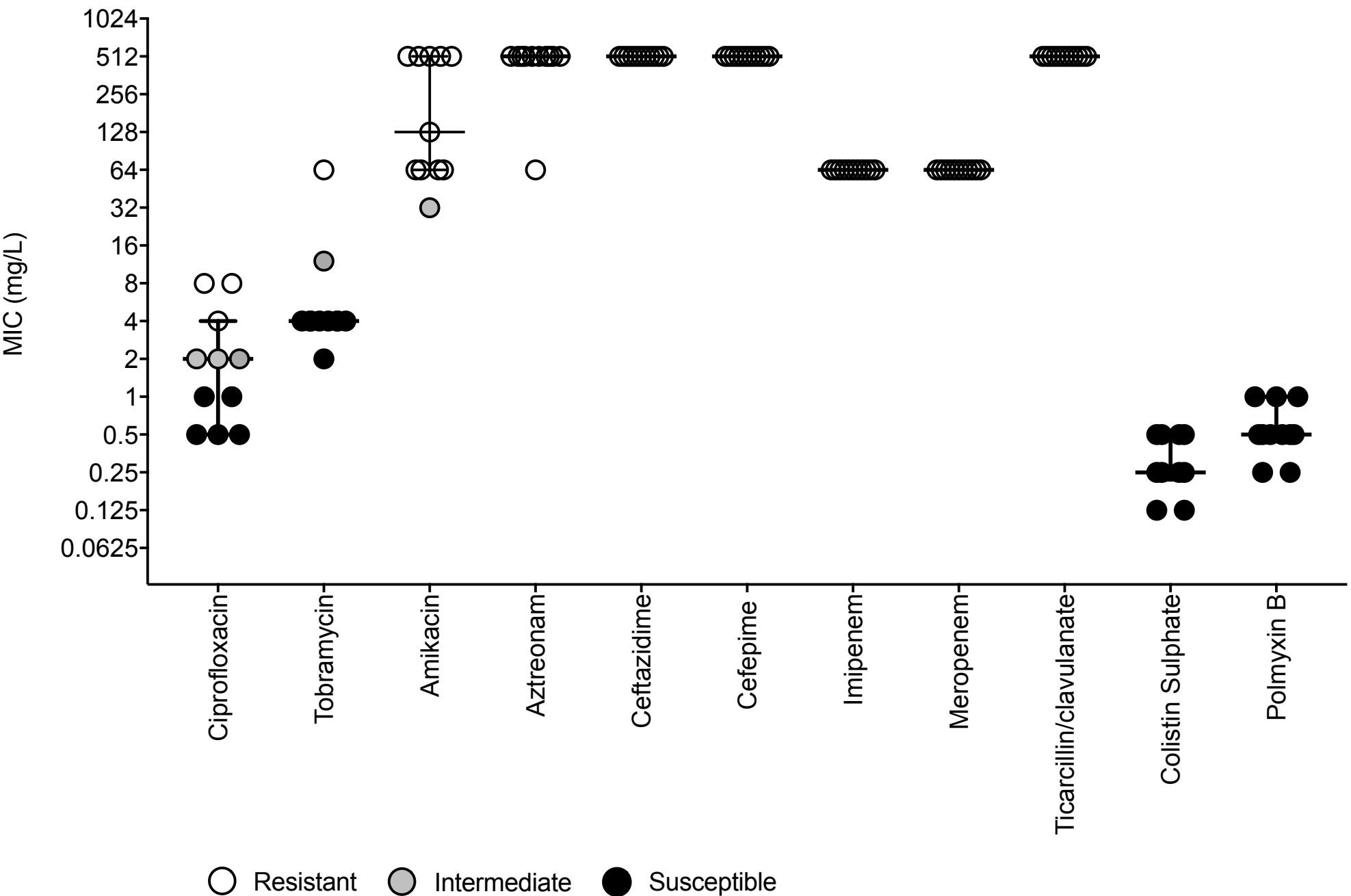

Supplement: S3 Fig — Isolates were categorized as resistant, intermediate or susceptible according to CLSI guidelines. The median and interquartile range are shown. Any isolates recorded as having a MIC greater than the maximum (>256 mg/L or >32 mg/L) value on the Etest® strip are shown as double the maximum concentration. (PDF) [file pone.0172179.s003.pdf]

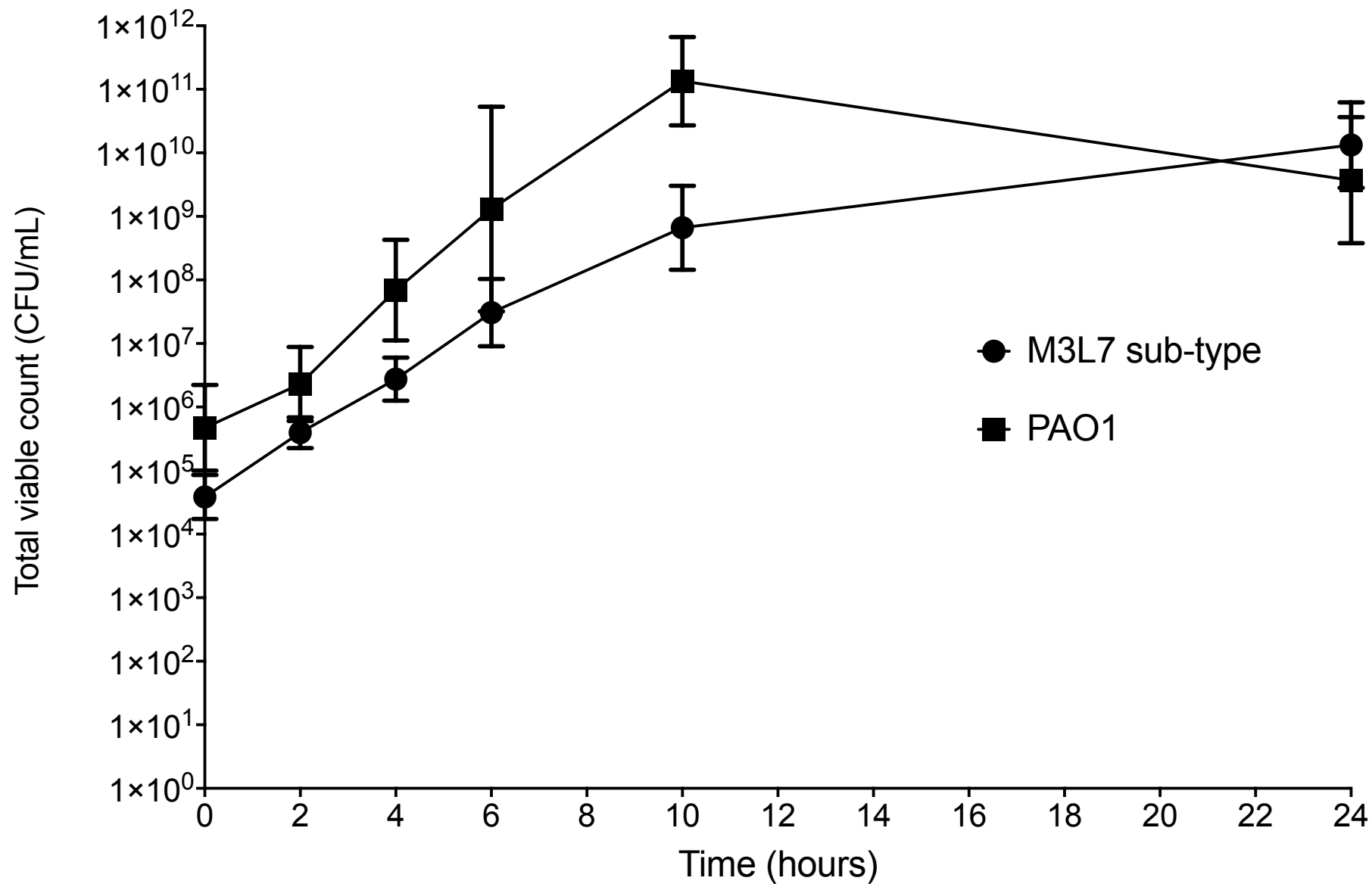

Supplement: S4 Fig — (PDF) [file pone.0172179.s004.pdf]
