# Supplementary material for: Within-host whole genome analysis of an antibiotic resistant Pseudomonas aeruginosa strain sub-type in cystic fibrosis
Source: PLoS One. 2017 Mar 8;12(3):e0172179. doi: 10.1371/journal.pone.0172179 (PMC5342179; doi:10.1371/journal.pone.0172179)
Supplement: S2 Table — (DOCX) [file pone.0172179.s006.docx]

**S2 Table. Literature search.**

| Chromosomally encoded protein  (locus tag of gene) | References | | |
| --- | --- | --- | --- |
|  | Antibiotic resistance | Hypermutation | Pathoadaptation |
| GyrA (PA3168) | [1] |  | [2,3] |
| GyrB (PA0004) | [1] |  | [2,3] |
| ParC (PA4964) | [1] |  |  |
| ParE (PA4967) | [1] |  |  |
| AmpC (PA4110) | [4-6] |  | [3,7] |
| AmpR (PA4109) | [5,6] |  | [3] |
| AmpE (PA4521) | [5] |  |  |
| AmpD (PA4522) | [5] |  |  |
| AmpP (PA4218) | [8] |  |  |
| AmpDh2 (PA5485) | [9] |  |  |
| AmpDh3 (PA0807) | [9] |  |  |
| AmpG (PA4393) | [8,10] |  |  |
| RpoN (PA4462) | [6] |  |  |
| PonA (PA5045) | [11] |  |  |
| MrcB (PA4700) | [12] |  |  |
| PbpA (PA4003) | [12] |  |  |
| FtsI (PA4418) | [12] |  |  |
| PbpC (PA2272) | [12] |  |  |
| DacB (PA3047) | [13] |  |  |
| DacC (PA3999) | [13] |  |  |
| PbpG (PA0869) | [13] |  |  |
| NagZ (PA3005) | [14] |  |  |
| CreB (PA0463) | [15] |  |  |
| CreC (PA0464) | [15] |  |  |
| CreD (PA0465) | [15] |  |  |
| PoxB (PA5297) | [16] |  |  |
| OprD (PA0958) | [17,18] |  | [2,3] |
| OXA-50 (PA5514) | [19] |  |  |
| Mpl (PA4020) | [20] |  | [2] |
| PIB-1 (PA5542) | [21] |  |  |
| Aph(3')-IIb (PA4119) | [22,23] |  |  |
| HpaA (PA4091) | [24] |  |  |
| DsbM (PA0058) | [25] |  |  |
| RplY (PA4671) | [26] |  |  |
| GalU (PA2023) | [26] |  |  |
| NuoA (PA2637) | [27] |  |  |
| NuoB (PA2638) | [27] |  |  |
| NuoD (PA2639) | [27] |  |  |
| NuoE (PA2640) | [27] |  |  |
| NuoF (PA2641) | [27] |  |  |
| NuoG (PA2642) | [26,27] |  |  |
| NuoH (PA2643) | [27] |  |  |
| NuoI (PA2644) | [27] |  |  |
| NuoJ (PA2645) | [27] |  |  |
| NuoK (PA2646) | [27] |  |  |
| NuoL (PA2647) | [27] |  |  |
| NuoM (PA2648) | [27] |  |  |
| NuoN (PA2649) | [27] |  |  |
| AmgS (PA5199) | [28] |  |  |
| PhoP (PA1179) | [29] |  |  |
| PhoQ PA1180) | [29] |  |  |
| PprA (PA4293) | [30] |  |  |
| PprB (PA4296) | [30] |  |  |
| MexA (PA0425) | [31,32] |  | [2] |
| MexB (PA0426) | [31,32] |  | [2,3] |
| MexC (PA4599) | [33] |  |  |
| MexD (PA4598) | [33] |  |  |
| MexE (PA2493) | [34] |  |  |
| MexF (PA2494) | [34] |  |  |
| MexH (PA4206) | [35] |  |  |
| MexI (PA4207) | [35] |  |  |
| MexJ (PA3677) | [36] |  |  |
| MexK (PA3676) | [36] |  |  |
| MexV (PA4374) | [37] |  |  |
| MexW (PA4375) | [37] |  |  |
| MexX (PA2019) | [32] |  |  |
| MexY (PA2018) | [32] |  | [3,7] |
| MuxA (PA2528) | [38] |  |  |
| MuxB (PA2527) | [38] |  |  |
| MuxC (PA2526) | [38] |  |  |
| OprJ (PA4597) | [33] |  |  |
| OprM (PA0427) | [31,32] |  |  |
| OprN (PA2495) | [34] |  |  |
| OpmB (PA2525) | [38] |  |  |
| OpmD (PA4208) | [35] |  |  |
| NalC (PA3721) | [39] |  |  |
| NalD (PA3574) | [40,41] |  | [2] |
| MexR (PA0424) | [31,39] |  | [2] |
| ArmR (PA3719) | [39] |  |  |
| MexS (PA2491) | [42,43] |  | [2] |
| MexT (PA2492) | [34,44] |  |  |
| MexZ (PA2020) | [45] |  | [2] |
| ArmZ (PA5471) | [26,46] |  |  |
| MexL (PA3678) | [36] |  |  |
| MexG (PA4205) | [12] |  |  |
| MvaT (PA4315) | [43] |  |  |
| NfxB (PA4600) | [33] |  | [2] |
| ParR (PA1799) | [46,47] |  |  |
| ParS (PA1798) | [46,47] |  |  |
| MutL (PA4946) |  | [48] | [3] |
| MutS (PA3620) |  | [48] |  |
| MutY (PA5147) |  | [48] |  |
| MutT (PA4400) |  | [48] |  |
| MutU (PA5443) |  | [48] |  |
| MutM (PA0357) |  | [48] |  |
| MucA (PA0763) |  |  | [2] |
| VgrG (PA5266) |  |  | [2] |
| AlgU (PA0762) |  |  | [2] |
| PA2099 |  |  | [2] |
| WbpM (PA3141) |  |  | [2] |
| BifA (PA4367) |  |  | [2] |
| MorA (PA4601) |  |  | [2] |
| DnaX (PA1532) |  |  | [2] |
| PcoA (PA2065) |  |  | [2] |
| Tle1 (PA3290) |  |  | [2] |
| BetT (PA5291) |  |  | [2] |
| RbDA (PA0861) |  |  | [2] |
| PA2455 |  |  | [2] |
| PA0977 |  |  | [2] |
| LasR (PA1430) |  |  | [2] |
| PelA (PA3064) |  |  | [2] |
| WspE (PA3704) |  |  | [2] |
| WspA (PA3708) |  |  | [2] |
| PhzB1 (PA4211) |  |  | [2] |
| PA4311 |  |  | [2] |
| RetS (PA4856) |  |  | [2] |
| AceE (PA5015) |  |  | [2] |
| AceF (PA5016) |  |  | [2] |
| PilQ (PA5040) |  |  | [2] |
| HtrB (PA3242) |  |  | [2] |
| YkoM (PA3458) |  |  | [2] |
| PilD (PA4528) |  |  | [2] |
| PdxY (PA5516) |  |  | [2] |
| YecS (PA0313) |  |  | [2] |
| PA1471 |  |  | [2] |
| PA1677 |  |  | [2] |
| PvdS (PA2426) |  |  | [2] |
| PA2490 |  |  | [2] |
| PA2602 |  |  | [2] |
| PA3222 |  |  | [2] |
| PA3939 |  |  | [2] |
| PA4642 |  |  | [2] |
| PA4963 |  |  | [2] |
| PhaF (PA5060) |  |  | [2] |
| PA5177 |  |  | [2] |
| CmpR (PA5437) |  |  | [2] |

**References**

1. Bruchmann S, Dotsch A, Nouri B, Chaberny IF, Haussler S. Quantitative contributions of target alteration and decreased drug accumulation to *Pseudomonas aeruginosa* fluoroquinolone resistance. Antimicrob Agents Chemother. 2013;57(3):1361-8.

2. Marvig RL, Sommer LM, Molin S, Johansen HK. Convergent evolution and adaptation of *Pseudomonas aeruginosa* within patients with cystic fibrosis. Nat Genet. 2015;47(1):57-64.

3. Marvig RL, Johansen HK, Molin S, Jelsbak L. Genome analysis of a transmissible lineage of *Pseudomonas aeruginosa* reveals pathoadaptive mutations and distinct evolutionary paths of hypermutators. PLoS Genet. 2013;9(9):e1003741.

4. Berrazeg M, Jeannot K, Ntsogo Enguene VY, Broutin I, Loeffert S, Fournier D, et al. Mutations in beta-Lactamase AmpC Increase Resistance of *Pseudomonas aeruginosa* Isolates to Antipseudomonal Cephalosporins. Antimicrob Agents Chemother. 2015;59(10):6248-55.

5. Juan C, Macia MD, Gutierrez O, Vidal C, Perez JL, Oliver A. Molecular mechanisms of beta-lactam resistance mediated by AmpC hyperproduction in *Pseudomonas aeruginosa* clinical strains. Antimicrob Agents Chemother. 2005;49(11):4733-8.

6. Caille O, Zincke D, Merighi M, Balasubramanian D, Kumari H, Kong KF, et al. Structural and functional characterization of *Pseudomonas aeruginosa* global regulator AmpR. J Bacteriol. 2014;196(22):3890-902.

7. Feliziani S, Marvig RL, Lujan AM, Moyano AJ, Di Rienzo JA, Krogh Johansen H, et al. Coexistence and within-host evolution of diversified lineages of hypermutable *Pseudomonas aeruginosa* in long-term cystic fibrosis infections. PLoS Genet. 2014;10(10):e1004651.

8. Kong KF, Aguila A, Schneper L, Mathee K. *Pseudomonas aeruginosa* beta-lactamase induction requires two permeases, AmpG and AmpP. BMC Microbiol. 2010;10:328.

9. Juan C, Moya B, Perez JL, Oliver A. Stepwise upregulation of the *Pseudomonas aeruginosa* chromosomal cephalosporinase conferring high-level beta-lactam resistance involves three AmpD homologues. Antimicrob Agents Chemother. 2006;50(5):1780-7.

10. Zhang Y, Bao Q, Gagnon LA, Huletsky A, Oliver A, Jin S, et al. *ampG* gene of *Pseudomonas aeruginosa* and its role in beta-lactamase expression. Antimicrob Agents Chemother. 2010;54(11):4772-9.

11. Handfield J, Gagnon L, Dargis M, Huletsky A. Sequence of the *ponA* gene and characterization of the penicillin-binding protein 1A of *Pseudomonas aeruginosa* PAO1. Gene. 1997;199(1-2):49-56.

12. http://www.pseudomonas.com

13. Ropy A, Cabot G, Sanchez-Diener I, Aguilera C, Moya B, Ayala JA, et al. Role of *Pseudomonas aeruginosa* low-molecular-mass penicillin-binding proteins in AmpC expression, beta-lactam resistance, and peptidoglycan structure. Antimicrob Agents Chemother. 2015;59(7):3925-34.

14. Asgarali A, Stubbs KA, Oliver A, Vocadlo DJ, Mark BL. Inactivation of the glycoside hydrolase NagZ attenuates antipseudomonal beta-lactam resistance in *Pseudomonas aeruginosa*. Antimicrob Agents Chemother. 2009;53(6):2274-82.

15. Zamorano L, Moya B, Juan C, Mulet X, Blazquez J, Oliver A. The *Pseudomonas aeruginosa* CreBC two-component system plays a major role in the response to beta-lactams, fitness, biofilm growth, and global regulation. Antimicrob Agents Chemother. 2014;58(9):5084-95.

16. Kong KF, Jayawardena SR, Del Puerto A, Wiehlmann L, Laabs U, Tummler B, et al. Characterization of *poxB*, a chromosomal-encoded *Pseudomonas aeruginosa* oxacillinase. Gene. 2005;358:82-92.

17. Sanbongi Y, Shimizu A, Suzuki T, Nagaso H, Ida T, Maebashi K, et al. Classification of OprD sequence and correlation with antimicrobial activity of carbapenem agents in *Pseudomonas aeruginosa* clinical isolates collected in Japan. Microbiol Immunol. 2009;53(7):361-7.

18. Kos VN, Deraspe M, McLaughlin RE, Whiteaker JD, Roy PH, Alm RA, et al. The resistome of *Pseudomonas aeruginosa* in relationship to phenotypic susceptibility. Antimicrob Agents Chemother. 2015;59(1):427-36.

19. Girlich D, Naas T, Nordmann P. Biochemical characterization of the naturally occurring oxacillinase OXA-50 of *Pseudomonas aeruginosa*. Antimicrob Agents Chemother. 2004;48(6):2043-8.

20. Tsutsumi Y, Tomita H, Tanimoto K. Identification of novel genes responsible for overexpression of *ampC* in *Pseudomonas aeruginosa* PAO1. Antimicrob Agents Chemother. 2013;57(12):5987-93.

21. Fajardo A, Hernando-Amado S, Oliver A, Ball G, Filloux A, Martinez JL. Characterization of a novel Zn(2)(+)-dependent intrinsic imipenemase from *Pseudomonas aeruginosa*. J Antimicrob Chemother. 2014;69(11):2972-8.

22. Hachler H, Santanam P, Kayser FH. Sequence and characterization of a novel chromosomal aminoglycoside phosphotransferase gene, *aph (3')-IIb*, in *Pseudomonas aeruginosa*. Antimicrob Agents Chemother. 1996;40(5):1254-6.

23. Hainrichson M, Yaniv O, Cherniavsky M, Nudelman I, Shallom-Shezifi D, Yaron S, et al. Overexpression and initial characterization of the chromosomal aminoglycoside 3'-O-phosphotransferase APH(3')-IIb from *Pseudomonas aeruginosa*. Antimicrob Agents Chemother. 2007;51(2):774-6.

24. Zeng L, Jin S. *aph(3')-IIb*, a gene encoding an aminoglycoside-modifying enzyme, is under the positive control of surrogate regulator HpaA. Antimicrob Agents Chemother. 2003;47(12):3867-76.

25. Wang X, Li M, Liu L, Mou R, Zhang X, Bai Y, et al. DsbM, a novel disulfide oxidoreductase affects aminoglycoside resistance in *Pseudomonas aeruginosa* by OxyR-regulated response. J Microbiol. 2012;50(6):932-8.

26. Islam S, Oh H, Jalal S, Karpati F, Ciofu O, Hoiby N, et al. Chromosomal mechanisms of aminoglycoside resistance in *Pseudomonas aeruginosa* isolates from cystic fibrosis patients. Clin Microbiol Infect. 2009;15(1):60-6.

27. Strateva T, Yordanov D. *Pseudomonas aeruginosa* - a phenomenon of bacterial resistance. J Med Microbiol. 2009;58(Pt 9):1133-48.

28. Lau CH, Fraud S, Jones M, Peterson SN, Poole K. Mutational activation of the AmgRS two-component system in aminoglycoside-resistant *Pseudomonas aeruginosa*. Antimicrob Agents Chemother. 2013;57(5):2243-51.

29. Wei Q, Tarighi S, Dotsch A, Haussler S, Musken M, Wright VJ, et al. Phenotypic and genome-wide analysis of an antibiotic-resistant small colony variant (SCV) of *Pseudomonas aeruginosa*. PLoS One. 2011;6(12):e29276.

30. Wang Y, Ha U, Zeng L, Jin S. Regulation of membrane permeability by a two-component regulatory system in *Pseudomonas aeruginosa*. Antimicrob Agents Chemother. 2003;47(1):95-101.

31. Poole K, Tetro K, Zhao Q, Neshat S, Heinrichs DE, Bianco N. Expression of the multidrug resistance operon *mexA-mexB-oprM* in *Pseudomonas aeruginosa*: *mexR* encodes a regulator of operon expression. Antimicrob Agents Chemother. 1996;40(9):2021-8.

32. Vettoretti L, Plesiat P, Muller C, El Garch F, Phan G, Attree I, et al. Efflux unbalance in *Pseudomonas aeruginosa* isolates from cystic fibrosis patients. Antimicrob Agents Chemother. 2009;53(5):1987-97.

33. Poole K, Gotoh N, Tsujimoto H, Zhao Q, Wada A, Yamasaki T, et al. Overexpression of the *mexC-mexD-oprJ* efflux operon in *nfxB*-type multidrug-resistant strains of *Pseudomonas aeruginosa*. Mol Microbiol. 1996;21(4):713-24.

34. Kohler T, Epp SF, Curty LK, Pechere JC. Characterization of MexT, the regulator of the MexE-MexF-OprN multidrug efflux system of *Pseudomonas aeruginosa*. J Bacteriol. 1999;181(20):6300-5.

35. Sekiya H, Mima T, Morita Y, Kuroda T, Mizushima T, Tsuchiya T. Functional cloning and characterization of a multidrug efflux pump, *mexHI-opmD*, from a *Pseudomonas aeruginosa* mutant. Antimicrob Agents Chemother. 2003;47(9):2990-2.

36. Chuanchuen R, Narasaki CT, Schweizer HP. The MexJK efflux pump of *Pseudomonas aeruginosa* requires OprM for antibiotic efflux but not for efflux of triclosan. J Bacteriol. 2002;184(18):5036-44.

37. Li Y, Mima T, Komori Y, Morita Y, Kuroda T, Mizushima T, et al. A new member of the tripartite multidrug efflux pumps, MexVW-OprM, in *Pseudomonas aeruginosa*. J Antimicrob Chemother. 2003;52(4):572-5.

38. Mima T, Kohira N, Li Y, Sekiya H, Ogawa W, Kuroda T, et al. Gene cloning and characteristics of the RND-type multidrug efflux pump MuxABC-OpmB possessing two RND components in *Pseudomonas aeruginosa*. Microbiology. 2009;155(Pt 11):3509-17.

39. Wilke MS, Heller M, Creagh AL, Haynes CA, McIntosh LP, Poole K, et al. The crystal structure of MexR from *Pseudomonas aeruginosa* in complex with its antirepressor ArmR. Proc Natl Acad Sci U S A. 2008;105(39):14832-7.

40. Morita Y, Cao L, Gould VC, Avison MB, Poole K. *nalD* encodes a second repressor of the *mexAB-oprM* multidrug efflux operon of *Pseudomonas aeruginosa*. J Bacteriol. 2006;188(24):8649-54.

41. Sobel ML, Hocquet D, Cao L, Plesiat P, Poole K. Mutations in PA3574 (*nalD*) lead to increased MexAB-OprM expression and multidrug resistance in laboratory and clinical isolates of *Pseudomonas aeruginosa*. Antimicrob Agents Chemother. 2005;49(5):1782-6.

42. Sobel ML, Neshat S, Poole K. Mutations in PA2491 (*mexS*) promote MexT-dependent *mexEF-oprN* expression and multidrug resistance in a clinical strain of *Pseudomonas aeruginosa*. J Bacteriol. 2005;187(4):1246-53.

43. Llanes C, Kohler T, Patry I, Dehecq B, van Delden C, Plesiat P. Role of the MexEF-OprN efflux system in low-level resistance of *Pseudomonas aeruginosa* to ciprofloxacin. Antimicrob Agents Chemother. 2011;55(12):5676-84.

44. Maseda H, Saito K, Nakajima A, Nakae T. Variation of the *mexT* gene, a regulator of the MexEF-oprN efflux pump expression in wild-type strains of *Pseudomonas aeruginosa*. FEMS Microbiol Lett. 2000;192(1):107-12.

45. Vogne C, Aires JR, Bailly C, Hocquet D, Plesiat P. Role of the multidrug efflux system MexXY in the emergence of moderate resistance to aminoglycosides among *Pseudomonas aeruginosa* isolates from patients with cystic fibrosis. Antimicrob Agents Chemother. 2004;48(5):1676-80.

46. Guenard S, Muller C, Monlezun L, Benas P, Broutin I, Jeannot K, et al. Multiple mutations lead to MexXY-OprM-dependent aminoglycoside resistance in clinical strains of *Pseudomonas aeruginosa*. Antimicrob Agents Chemother. 2014;58(1):221-8.

47. Muller C, Plesiat P, Jeannot K. A two-component regulatory system interconnects resistance to polymyxins, aminoglycosides, fluoroquinolones, and beta-lactams in *Pseudomonas aeruginosa*. Antimicrob Agents Chemother. 2011;55(3):1211-21.

48. Oliver A, Mena A. Bacterial hypermutation in cystic fibrosis, not only for antibiotic resistance. Clin Microbiol Infect. 2010;16(7):798-808.
