# Supplementary material for: Within-host whole genome analysis of an antibiotic resistant Pseudomonas aeruginosa strain sub-type in cystic fibrosis
Source: PLoS One. 2017 Mar 8;12(3):e0172179. doi: 10.1371/journal.pone.0172179 (PMC5342179; doi:10.1371/journal.pone.0172179)
Supplement: S3 Table — (DOCX) [file pone.0172179.s007.docx]

**S3 Table. General genome features of isolates within the M3L7 sub-type.**

| Isolate ID | Accession number | Length (bp) | Length (contigs > 1K) | GC content | Contigs  (> 1Kbp) | Total contigs | n50 |
| --- | --- | --- | --- | --- | --- | --- | --- |
| AUS959 | ERS1245422 | 6233689 | 6219168 | 66.5 | 54 | 96 | 347242 |
| AUS960 | ERS1245423 | 6241265 | 6214198 | 66.5 | 53 | 120 | 341833 |
| AUS961 | ERS1245424 | 6198826 | 6172544 | 66.5 | 53 | 119 | 347366 |
| AUS962 | ERS1245425 | 6200577 | 6175555 | 66.5 | 50 | 113 | 412957 |
| AUS963 | ERS1245426 | 6234304 | 6216242 | 66.5 | 53 | 101 | 405954 |
| AUS964 | ERS1245427 | 6234659 | 6216370 | 66.5 | 64 | 113 | 229847 |
| AUS965 | ERS1245428 | 6233068 | 6221619 | 66.5 | 66 | 103 | 267761 |
| AUS966 | ERS1245429 | 6237308 | 6216084 | 66.5 | 70 | 129 | 245225 |
| AUS967 | ERS1245430 | 6240999 | 6214162 | 66.5 | 60 | 126 | 287620 |
| AUS968 | ERS1245431 | 6236275 | 6216231 | 66.5 | 78 | 133 | 173428 |
| AUS969 | ERS1245432 | 6236732 | 6217235 | 66.5 | 63 | 113 | 245224 |
