# Supplementary material for: Within-host whole genome analysis of an antibiotic resistant Pseudomonas aeruginosa strain sub-type in cystic fibrosis
Source: PLoS One. 2017 Mar 8;12(3):e0172179. doi: 10.1371/journal.pone.0172179 (PMC5342179; doi:10.1371/journal.pone.0172179)
Supplement: S1 File — (DOCX) [file pone.0172179.s008.docx]

**S1File**

**Collection and processing of sputum samples**

Sputum samples were stored at 4 °C and processed within 12 hours of collection. Each sputum sample was treated with an equal volume or double the volume of Sputasol (Thermo Scientific^TM^), depending on sputum viscosity, incubated at 35 °C for 20 mins and homogenised by vortexing for at least 60 s. Serial ten-fold dilutions of the subsequent suspension were prepared and evenly inoculated onto chocolate agar with bacitracin followed by incubation at 36 °C for up to 36 hours.

**Allele-specific PCR for AUST-02**

Each isolate initially underwent PCR screening for AUST-02 and non-AUST-02 *P. aeruginosa* strains using primers listed in Table 1. Briefly, these primers were used to distinguish a SNP present on the *mexZ* gene that is associated with the AUST-02 strain. The reverse primer (Con_Reverse) targeted conserved sequences across all *P. aeruginosa* strains, whereas the forward primers were designed to detect AUST-02 (AUST-02_Forward) or Non-AUST-02 (Non-AUST-02_Forward) strains.

**Table 1. Primers use to screen for AUST-02 and non-AUST-02 *P. aeruginosa* strains.**

| Primer name | Primer sequence (5’-3’) |
| --- | --- |
| AUST-02_Forward | GCGCCTTCGGCCAGACT |
| Non-AUST-02_Forward | GCGCCTTCGGCCAGACC |
| Con_Reverse | CTGGCGTTTTCGTCGGGTA |

Two reaction mixes were used. The AUST-02 reaction comprised the Con_Reverse and AUST-02_Forward primers, and the Non-AUST-02 reaction comprised the Con_Reverse and Non-AUST-02_Forward primers. Each reaction mix contained 10 µL SYBR PCR mix (Invitrogen), 0.5 µM of forward and reverse primers, 2 µL of heat-denatured isolate DNA (prepared as per [1]) and DNase-free water made up to a total volume of 20 µL. The PCR amplification was performed on a Rotor-Gene Q instrument (QIAGEN Pty Ltd) with the following conditions: an initial enzyme activation step at 95 °C for 2 mins, followed by 40 cycles of two-step cycling (denaturation at 95 °C for 15 s, and cycling at 60 ^o^C for 30 s, with fluorescent signal acquired at the latter 60 ^o^C step). A reaction was classified as AUST-02 positive when the cycle threshold [Ct] value was <30 cycles in the AUST-02 reaction and was lower (by approximately 10 cycles) than the Ct value in the Non-AUST-02 reaction. A reaction was classified as Non-AUST-02 positive when the Ct value was <30 cycles in the Non-AUST-02 reaction and was lower (by approximately 10 cycles) than the Ct value in the AUST-02 reaction.

Isolates that provided Ct values ≥ 30 cycles in both the AUST-02 and Non-AUST-02 reactions were classed as non-*P. aeruginosa* (in this study, *n*=6). Isolates that tested positive for a non-AUST-02 *P. aeruginosa* strain underwent Sequenom iPLEX SNP-based strain typing to determine the strain [2].

**References**

1. Anuj SN, Whiley DM, Kidd TJ, Bell SC, Wainwright CE, Nissen MD, et al. Identification of *Pseudomonas aeruginosa* by a duplex real-time polymerase chain reaction assay targeting the *ecfX* and the *gyrB* genes. Diagn Microbiol Infect Dis 2009; 63(2):127-31.

2. Syrmis MW, Kidd TJ, Moser RJ, Ramsay KA, Gibson KM, Anuj S, et al. A comparison of two informative SNP-based strategies for typing *Pseudomonas aeruginosa* isolates from patients with cystic fibrosis. BMC Infect Dis. 2014;14:307.
