# Supplementary material for: Within-host whole genome analysis of an antibiotic resistant Pseudomonas aeruginosa strain sub-type in cystic fibrosis
Source: PLoS One. 2017 Mar 8;12(3):e0172179. doi: 10.1371/journal.pone.0172179 (PMC5342179; doi:10.1371/journal.pone.0172179)
Supplement: S2 File — (DOCX) [file pone.0172179.s009.docx]

**S2 File**

Data sets containing individual data points relating to S3 Fig and to S4 Fig.

**Table 1:** **Minimum inhibitory concentrations of M3L7 isolates against 11 anti-pseudomonal antibiotics.**

|  | Minimum inhibitory concentration (mg/L) | | | | | | | | | | |
| --- | --- | --- | --- | --- | --- | --- | --- | --- | --- | --- | --- |
| Isolate ID | Ciprofloxacin | Tobramycin | Amikacin | Aztreonam | Ceftazidime | Cefepime | Imipenem | Meropenem | Ticarcillin/clavulanate | Colistin sulphate | Polymyxin B |
| AUS959 | 0.5 | 4 | >256 | >256 | >256 | >256 | >32 | >32 | >256 | 0.25 | 0.25 |
| AUS960 | 1 | 4 | 64 | >256 | >256 | >256 | >32 | >32 | >256 | NT | 0.5 |
| AUS961 | 2 | 64 | >256 | >256 | >256 | >256 | >32 | >32 | >256 | 0.5 | 0.5 |
| AUS962 | 2 | 4 | 128 | 64 | >256 | >256 | >32 | >32 | >256 | 0.5 | 1 |
| AUS963 | 8 | 2 | 32 | >256 | >256 | >256 | >32 | >32 | >256 | 0.25 | 0.5 |
| AUS964 | 4 | 4 | 64 | >256 | >256 | >256 | >32 | >32 | >256 | 0.125 | 0.5 |
| AUS965 | 0.5 | 4 | >256 | >256 | >256 | >256 | >32 | >32 | >256 | 0.25 | 0.5 |
| AUS966 | 8 | 12 | >256 | >256 | >256 | >256 | >32 | >32 | >256 | 0.25 | 0.5 |
| AUS967 | 1 | 4 | 64 | >256 | >256 | >256 | >32 | >32 | >256 | 0.5 | 1 |
| AUS968 | 0.5 | 4 | >256 | >256 | >256 | >256 | >32 | >32 | >256 | 0.125 | 0.25 |
| AUS969 | 2 | 4 | 64 | >256 | >256 | >256 | >32 | >32 | >256 | 0.5 | 1 |

NT: not tested

**Table 2. Total viable counts of the M3L7 sub-type and PAO1 grown in Luria-Bertani Broth.**

|  | Isolate (CFU/mL) | | | | | | | | | | | | | |
| --- | --- | --- | --- | --- | --- | --- | --- | --- | --- | --- | --- | --- | --- | --- |
| Time (hours) | AUS959 | AUS960 | AUS961 | AUS962 | AUS963 | AUS964 | AUS965 | AUS966 | AUS967 | AUS968 | AUS969 | PAO1 | PAO1 | PAO1 |
| 0 | 5.50E+04 | 3.70E+04 | 5.20E+04 | 6.70E+04 | 7.00E+04 | 1.10E+04 | 3.00E+03 | 1.12E+05 | 1.80E+04 | 3.40E+04 | 2.40E+05 | 2.40E+05 | 8.20E+05 | 5.40E+05 |
| 2 | 7.50E+05 | 4.20E+05 | 6.70E+05 | 7.90E+05 | 3.70E+05 | 4.70E+05 | 3.80E+04 | 4.80E+05 | 3.60E+05 | 3.60E+05 | 5.40E+05 | 3.80E+06 | 2.50E+06 | 1.30E+06 |
| 4 | 2.00E+06 | 9.50E+06 | 5.10E+06 | 8.90E+06 | 2.70E+06 | 3.10E+06 | 1.30E+05 | 2.80E+06 | 4.60E+06 | 1.40E+06 | 4.00E+06 | 1.61E+08 | 4.80E+07 | 4.30E+07 |
| 6 | 9.00E+06 | 2.80E+08 | 4.80E+07 | 7.50E+07 | 1.90E+07 | 3.40E+07 | 3.70E+05 | 4.20E+07 | 3.20E+08 | 2.20E+07 | 3.40E+07 | 5.74E+09 | 1.33E+09 | 2.90E+08 |
| 10 | 2.90E+08 | 5.17E+09 | 7.90E+08 | 2.48E+09 | 7.80E+08 | 1.36E+09 | 1.29E+06 | 1.14E+09 | 4.51E+09 | 3.30E+08 | 1.61E+09 | 6.76E+10 | 1.48E+11 | 2.43E+11 |
| 24 | 4.35E+10 | 7.48E+10 | 1.89E+10 | 5.46E+10 | 2.60E+09 | 3.60E+10 | 4.10E+07 | 4.82E+10 | 8.63E+10 | 1.83E+09 | 2.41E+10 | 1.07E+10 | 2.40E+09 | 2.00E+09 |
